# Debrief to Development: Creating a Teaching Session on ‘Recognising Overdose’

**DOI:** 10.1192/bjo.2026.11344

**Published:** 2026-06-30

**Authors:** Isabel Barnett, Andrew Hayward, Charles Pope

**Affiliations:** Leeds and York Partnership NHS Foundation Trust, Leeds, United Kingdom

## Abstract

**Aims:**

Following a serious untoward incident (SUI) involving a service user under the care of the Intensive Home Treatment Team for Older People (IHTT) in Leeds, a learning need was identified around the recognition of medication overdose, particularly in community mental health settings. Debrief around the incident highlighted a gap in confidence and awareness, particularly among non-medical staff in recognising potential overdose presentations outside acute hospital environments.

To address this, we designed and implemented a targeted teaching session with the aim of strengthening clinical vigilance and early identification of possible overdose and provide practical, scenario-based education for frontline staff, to ultimately reduce the risk of similar incidents by improving knowledge, confidence and decision-making.

**Methods:**

A teaching session was developed by resident doctors in response to the SUI learning. It was designed for delivery to non-medical, community-based mental health staff and focused on recognising overdose presentations, including atypical or non-specific symptoms. Teaching sessions were delivered across a 1-hour format and incorporated case-based discussion, practical scenarios, and guidance around escalation and response. Feedback was collected through online forms in addition to informal verbal and email responses. Quantitative and qualitative analyses were performed to evaluate the quality, relevance andperceived impact of the teaching.

**Results:**

More than 50 staff members have attended the sessions so far, with more sessions planned. Quantitative analysis showed high levels of satisfaction and perceived benefits: 100% of respondents rated the quality, clarity and relevance of the session as 5/5. Participants reported a significant improvement in understanding of overdose presentations (mean score 4.83/5) and 94% reported increased confidence in responding to suspected overdose situations (mean score 4.39/5). Qualitative feedback highlighted strong engagement with the session content, with particular value placed on worked case examples. Suggestions for improvement included covering additional scenarios, proving an accessible quick-reference document and shortening the session to allow wider staff engagement.

**Conclusion:**

This project successfully addressed an identified learning gap following an SUI and was positively received by community mental health staff. Early feedback suggests it improves confidence and awareness in recognising potential overdose presentations in community settings. Future plans include wider and sustained implementation through a concise 15-minute core teaching package, development of a quick-reference guide, and further delivery to additional community teams, as well as inpatient units that have expressed an interest.

The initiative demonstrates how structured, reflective learning from adverse events can translate into meaningful improvements in frontline clinical practice.

